# Catastrophic Health Expenditure Associated With Frailty in Community-Dwelling Chinese Older Adults: A Prospective Cohort Analysis

**DOI:** 10.3389/fpubh.2021.718910

**Published:** 2021-09-09

**Authors:** Lijun Fan, Xiang-Yu Hou, Yingyan Liu, Sunan Chen, Qian Wang, Wei Du

**Affiliations:** ^1^School of Public Health, Southeast University, Nanjing, China; ^2^School of Health and Wellbeing, University of Southern Queensland, Toowoomba, QLD, Australia; ^3^Guangdong Provincial Geriatrics Institute, Guangdong Provincial People's Hospital, Guangdong Academy of Medical Sciences, Guangzhou, China

**Keywords:** catastrophic health expenditure, frailty, older people, longitudinal, China

## Abstract

**Background:** Catastrophic health expenditure (CHE) represents a key indicator for excessive financial burden due to out-of-pocket (OOP) healthcare costs, which could push the household into poverty and is highly pronounced in households with members at an advanced age. Previous studies have been devoted to understanding the determinants for CHE, yet little evidence exists on its association with frailty, an important geriatric syndrome attracting growing recognition. We thus aim to examine the relationship between frailty and CHE and to explore whether this effect is moderated by socioeconomic-related factors.

**Methods:** A total of 3,277 older adults were drawn from two waves (2011 and 2013) of the China Health and Retirement Longitudinal Study (CHARLS). CHE was defined when OOP healthcare expenditure exceeded a specific proportion of the capacity of the household to pay. Frailty was measured following the Fried Phenotype (FP) scale. Mixed-effects logistic regression models were employed to assess the longitudinal relationship between frailty and CHE, and stratification analyses were conducted to explore the moderation effect.

**Results:** The incidence of CHE among Chinese community-dwelling older adults was 21.76% in 2011 and increased to 26.46% in 2013. Compared with non-frail individuals, prefrail or frail adults were associated with higher odds for CHE after controlling for age, gender, residence, education, marriage, income, health insurance, smoking, drinking, and comorbidity (prefrail: odds ratio (OR) = 1.32, 95%CI = 1.14–1.52; frail: OR = 1.67, 95%CI = 1.13–2.47). Three frailty components including weakness, exhaustion, and shrinking contributed to a significantly increased likelihood of CHE (all *p* < 0.05), while the other two components including slowness and inactivity showed a non-significant effect (all *p* > 0.05). Similar effects from frailty on CHE were observed across socioeconomic-related subgroups differentiated by gender, residence, education, household income, and social health insurance.

**Conclusions:** Frailty is a significant predictor for CHE in China. Developing and implementing cost-effective strategies for the prevention and management of frailty is imperative to protect households from financial catastrophe.

## Introduction

A considerable number of individuals are confronted with a huge economic burden due to out-of-pocket (OOP) healthcare expenditures worldwide, and consequently, often place their families under a situation of unanticipated financial catastrophe or impoverishment (1). Although health insurance arrangements in many countries including China have achieved unprecedented progress in recent decades, particularly by expanding medical insurance coverage and increasing reimbursement benefits, their role in protecting the individuals or households from being pushed into poverty remains a challenge (2, 3). To help quantify and deal with the financial difficulties of households resulting from healthcare costs, researchers have generally agreed on a term called “catastrophic health expenditure (CHE),” which is defined as if healthcare spending exceeds a specified level of tolerance or threshold from the capacity of the household to pay (4, 5). The occurrence of CHE could absorb a large proportion of the household budget and leads to the sacrifice of the consumption of daily necessities, thereby affecting household living conditions and further deteriorating individual well-being (4, 5). Meanwhile, CHE is highly pronounced among older people, who account for the high demand for healthcare services but have limited income (3, 6). Along with the rapid and dramatic population aging, it is thus imperative to understand the CHE prevalence and its associated risk factors among elderly people.

Frailty, increasingly known as a good proxy for biological aging, is a multidimensional geriatric syndrome characterized by decreased resilience to stressors due to a generalized decline or age-related health deficits across multiple physiological systems (7, 8). In China, the first study using a nationally representative sample to estimate frailty prevalence found that frailty affected a large proportion of about 7.0% of the community-dwelling older people (9), and a further meta-analysis reported the prevalence of frailty to vary from 5.9 to 17.4% (10). Frailty, similar to many other geriatric assessments, was shown to predict adverse health outcomes and poor quality of life of older people (11, 12). In addition, previous studies demonstrated that frailty could predict poor recovery from stressors (13), as well as increased healthcare utilization and costs (14–17). For example, one Chinese study by Xu et al. (13) revealed the connection between frailty and poor recovery from activities of daily living (ADL) disability among non-disabled community-dwelling older adults (13). A recent meta-analysis demonstrated that healthcare costs increased by $79–13,423.83 in the prefrail elderly and by $616–32,549.96 in the frail elderly than the robust community-dwelling individuals, based on seven cohort studies comprised of 3,750,611 participants (17). Evidence from China also documented that frailty was an independent determinant of primarily increased outpatient and self-treatment expenditure among older adults (16).

However, an examination into solely healthcare costs seems inadequate to reveal the accurate economic burden exerted on individuals and households, considering that the same amount of healthcare spending may mean a different story for financially deprived or affluent families. A more comprehensive understanding of the disease burden can be gained if the impact of frailty on CHE is clear, which, however, remains to be elucidated. To date, there has been minimal research investigating the association between frailty and CHE, which yet concluded inconsistent findings and were limited in their cross-sectional design (18, 19). To fill in the research gap, the present study employs a national dataset to examine the longitudinal relationship between frailty and CHE among the community-dwelling older adults in China, as well as to explore whether this effect is moderated by socioeconomic-related factors to figure out subgroup inequities.

## Materials and Methods

### Data and Sample

The study participants were drawn from two waves (2011 and 2013) of the China Health and Retirement Longitudinal Study (CHARLS), a nationally representative longitudinal survey of the middle-aged and older Chinese population. A detailed description of the CHARLS survey has been reported elsewhere (20). Briefly, samples in CHARLS were selected using the multistage stratified sampling design and probability proportional sampling technique from 150 county-level units in 28 provinces of China. The participant information was collected *via* face-to-face computer-assisted interviews using structured questionnaires, which contained a wide range of data such as sociodemographics, family structure, health, biomarkers, health use, and expenditure. The CHARLS team carried out the baseline survey in 2011, which recruited ~17,000 participants, and held follow-up surveys biennially in 2013, 2015, and 2018.

In this study, we limited the sample to older adults aged adults 60 years or above in 2011 (*N* = 7,423) and included only the previous two waves of the survey due to the lack of key variables used for constructing frailty in the two latter waves. After excluding participants who did not complete the follow-up survey in 2013 (*N* = 1,233) or reported missing information on the study variables (*N* = 2,913), a final sample of 3,277 subjects were eligible for analysis in this study, and study variables for each participant were repeatedly measured at every available time point in both 2011 and 2013. The number of missing values was primarily due to incomplete frailty assessment, which was not unanticipated because the question about the frailty component of inactivity was originally designed in CHARLS protocol to be administered in only a random subsample of half of the study participants. The CHARLS protocol was approved by the Ethics Review Committee of Peking University (No.: IRB00001052-11015), and all participants provided written informed consent at the time of enrollment.

### Measurements

#### Frailty

Frailty was measured following the Fried frailty phenotype (FP) scale, a tool that had been rigorously validated previously, in which five items were assessed: slowness, weakness, exhaustion, inactivity, and shrinking (9, 14, 21). Slowness was defined if gait speed, measured using the average of two trials of walk tests over a 2.5 m course, was at or below the 20th percentile of the gender- and height-adjusted population distribution. The criteria for weakness were met when the maximum handgrip strength was below or equal to the 20th percentile of the population distribution, after adjusting for gender and body mass index (BMI). Exhaustion was defined when the answer to the two questions from the modified Centre for Epidemiological Studies-Depression (CES-D) scale (“I could not get going,” “I felt everything I did was an effort”) was “occasionally or a moderate amount of the time” or “most of the time.” Participants met the criteria for inactivity if they answered “no” to the question of “during a usual week, did you walk for at least 10 min continuously?” Respondents met the shrinking criteria when they currently had a BMI ≤ of 18.5 kg/m^2^ or self-reported loss of at least 5 kg in the previous year. Frailty was treated as missing if two or more frailty components were unavailable for each individual. We classified participants into different frailty levels according to previous literature (9, 14, 21), in which individuals fulfilling none of the five criteria were considered as “non-frail,” one or two criteria as “prefrail,” and three or more criteria as “frail.”

#### Catastrophic Health Expenditure

Catastrophic health expenditure was defined when OOP payment for healthcare was matched or exceeded a specified proportion of the capacity of the household to pay (4). In this study, we defined CHE as “yes” if OOP health spending was equal to or higher than 40% of the total non-food expenditure of households, in accordance with most previous studies to facilitate comparisons (2, 22). The numerator was OOP healthcare expenditure, which was calculated by summing up the self-reported medical OOP expenditure of respondents and their spouses on outpatient and inpatient care in the last year. In this study, annual outpatient OOP expenditure was evaluated by multiplying the self-reported monthly OOP outpatient payments by 12 to get the whole-year estimate, and annual inpatient OOP expenditure was assessed by self-report of participants of OOP payments for inpatient visits in the past year. The denominator was the total annual non-food expenditure of the household as a proxy for capacity to pay, which was obtained by deducting the annual food-based spending of the household from the total annual consumption expenditure. CHE was a binary variable, indicating whether or not the household of the participant had catastrophic healthcare spending.

#### Covariates

The following variables were considered as potential covariates in this study: age, gender, place of residence (rural and urban), educational attainment (no former education or illiterate, literate but did not finish primary school, primary school, and middle school and above), marital status (married and others), annual household income per capita in quartiles, social health insurance (none, New Rural Cooperative Medical Scheme, Urban Employee Basic Medical Insurance, Urban Resident Basic Medical Insurance, and others), smoking behavior (never smoked, former smoker, and current smoker), drinking habit (never drunk, drink but less than one time per month, and drink more than one time per month), and physical comorbidity with the presence of two or more noncommunicable chronic diseases (no and yes).

### Statistical Analysis

All statistical analyses were performed using STATA software version 16.0 (StataCorp, College Station, TX). Two-tailed *p* < 0.05 were considered statistically significant. The baseline characteristics of study participants according to frailty status were descriptively summarized with numbers and percentages and were statistically compared using the χ^2^-test. CHE incidence was calculated as the percentage of individuals incurring CHE during a certain period. McNemar's Chi-square test was conducted to compare the CHE incidence between 2 years in the total sample, and the Chi-square test for independent samples was carried out to examine whether CHE incidence differed by frailty status in each year.

To take the correlated data into account, a panel data approach of mixed-effects logistic regression models was performed to explore the longitudinal effect of frailty on CHE, and the results were expressed as odds ratio (OR) and 95% CI. Five models were hierarchically conducted to account for the potential confounding. Model 1, a crude model without adjustment for any covariates; Model 2, adjusting for covariates including age and gender; Model 3, additionally adjusting for residence, education, marital status, income, and health insurance; Model 4, additionally adjusting for smoking, and drinking; and Model 5, additionally adjusting for physical comorbidity. We further conducted subgroup analyses stratified by major socioeconomic-related factors including gender, place of residence, educational attainment, household income level, and social health insurance, using the same mixed-effects logistic regression but with the stratification variable removed from the model. The likelihood-ratio test was used to explore whether the interaction effect was significant.

Sensitivity analyses were undertaken by using different thresholds for classifying CHE according to the definitions of WHO and World Bank (4). CHE was defined in alternative ways, i.e., if OOP healthcare expenditure matched or exceeded 10 and 25% of the total household consumption expenditure, and 25% of the total non-food household consumption expenditure (4).

## Results

### Baseline Sample Characteristics

The study participants had a mean (SD) age of 66.95 (5.67) years and 52.33% of them were men. Table 1 shows their baseline characteristics according to frailty status. The participants with older age, rural residence, no health insurance, less education, non-married status, more deprived household, and physical comorbidity were relatively more susceptible to frailty (all *p* < 0.05). The frailty status between men and women was not found significantly different in our sample, and thus were the individuals with different smoking or drinking behaviors (all *p* > 0.05).

**Table 1:** Baseline characteristics of study participants according to frailty status.

**Characteristics**	**Overall (*n =* 3,277)**	**No. (%) of participants by frailty status**			***p*** **-value^a^**
		**Non-frail (***n =*** 1,706)**	**Pre-frail (***n =*** 1,479)**	**Frail (***n =*** 92)**	
Age					<0.001
60–64	1,392 (42.48)	819 (48.01)	555 (37.53)	18 (19.57)	
65–69	922 (28.14)	500 (29.31)	396 (26.77)	26 (28.26)	
70–74	570 (17.39)	261 (15.30)	284 (19.20)	25 (27.17)	
75–79	299 (9.12)	103 (6.04)	183 (12.37)	13 (14.13)	
≥80	94 (2.87)	23 (1.35)	61 (4.12)	10 (10.87)	
Gender					0.846
Male	1,715 (52.33)	886 (51.93)	782 (52.87)	47 (51.09)	
Female	1,562 (47.67)	820 (48.07)	697 (47.13)	45 (48.91)	
Residence					<0.001
Rural	2,656 (81.05)	1,327 (77.78)	1,246 (84.25)	83 (90.22)	
Urban	621 (18.95)	379 (22.22)	233 (15.75)	9 (9.78)	
Educational attainment					<0.001
No formal education or illiterate	1,113 (33.96)	514 (30.13)	557 (37.66)	42 (45.65)	
Literate but did not finish primary school	716 (21.85)	377 (22.10)	320 (21.64)	19 (20.65)	
Primary school	880 (26.85)	486 (28.49)	374 (25.29)	20 (21.74)	
Middle school and above	568 (17.33)	329 (19.28)	228 (15.42)	11 (11.96)	
Marital status					<0.001
Married	2,669 (81.45)	1,433 (84.00)	1,170 (79.11)	66 (71.74)	
Others	608 (18.55)	273 (16.00)	309 (20.89)	26 (28.26)	
Household income per capita					<0.001
Quartile 1 (deprived)	857 (26.15)	369 (21.63)	450 (30.43)	38 (41.30)	
Quartile 2	877 (26.76)	441 (25.85)	409 (27.65)	27 (29.35)	
Quartile 3	859 (26.21)	473 (27.73)	368 (24.88)	18 (19.57)	
Quartile 4 (affluent)	684 (20.87)	423 (24.79)	252 (17.04)	9 (9.78)	
Health insurance					<0.001
None	174 (5.31)	90 (5.26)	77 (5.21)	7 (7.61)	
New rural cooperative medical scheme	2,542 (77.57)	1,276 (74.79)	1,186 (80.19)	80 (86.96)	
Urban employee basic medical insurance	294 (8.97)	187 (10.96)	105 (7.10)	2 (2.17)	
Urban resident basic medical insurance	164 (5.00)	94 (5.51)	68 (4.60)	2 (2.17)	
Other insurances	103 (3.14)	59 (3.46)	43 (2.91)	1 (1.09)	
Smoking					0.371
Never	1,841 (56.18)	982 (57.56)	814 (55.04)	45 (48.91)	
Former	370 (11.29)	186 (10.90)	172 (11.63)	12 (13.04)	
Current	1,066 (32.53)	538 (31.54)	493 (33.33)	35 (38.04)	
Drinking					0.060
Never	2,253 (68.75)	1,142 (66.94)	1,043 (70.52)	68 (73.91)	
Drink but less than once per month	201 (6.13)	113 (6.62)	80 (5.41)	8 (8.70)	
Drink more than once per month	823 (25.11)	451 (26.44)	356 (24.07)	16 (17.39)	
Physical comorbidity					<0.001
No	1,900 (57.98)	1,047 (61.37)	808 (54.63)	45 (48.91)	
Yes	1,377 (42.02)	659 (38.63)	671 (45.37)	47 (51.09)	

a *p-values were calculated from chi-square test for categorical variables*.

**Figure 1:**
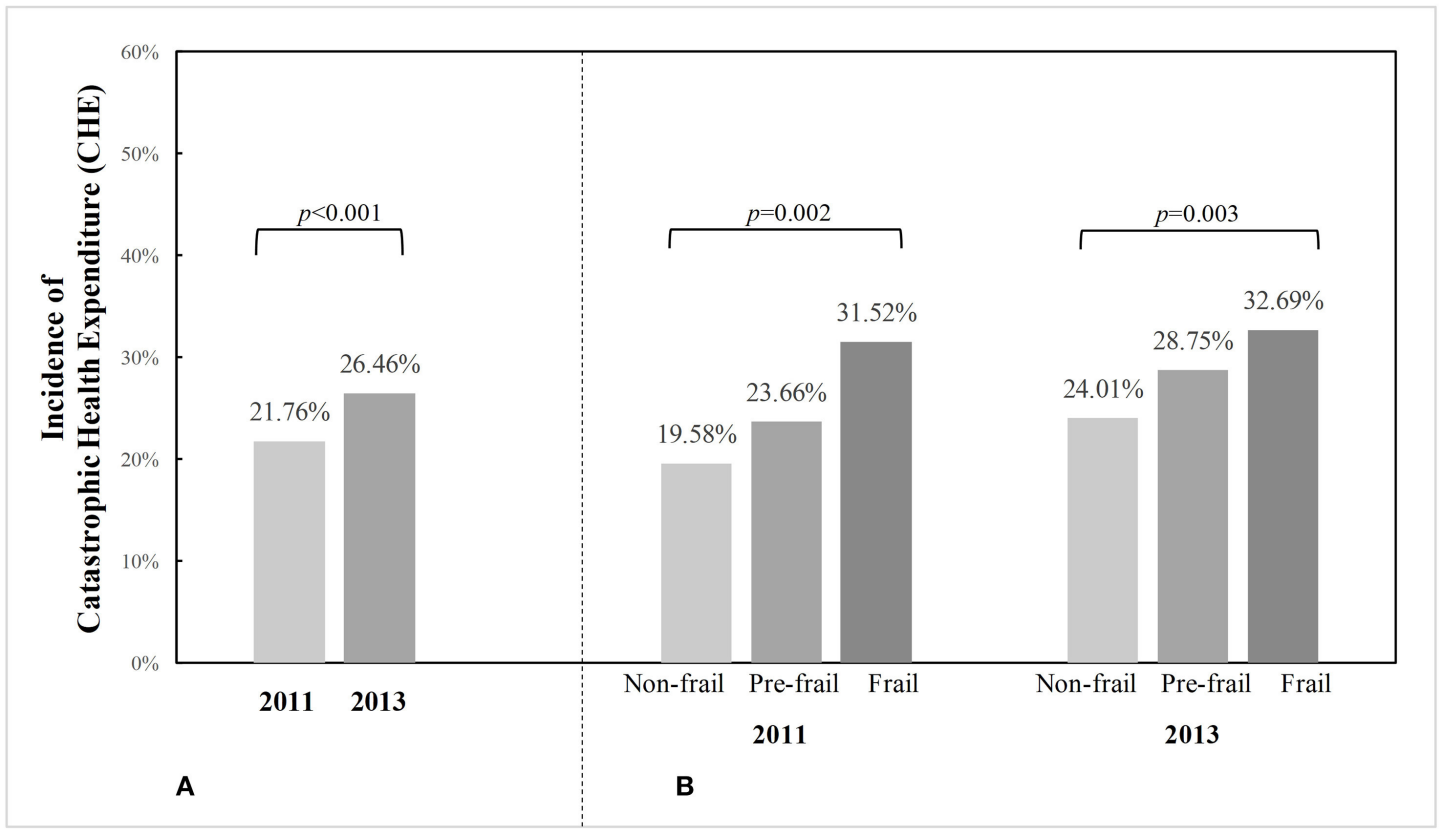
Incidence of catastrophic health expenditure (CHE) among Chinese community-dwelling older adults from 2011 to 2013, in the total sample and by frailty status. *p*-values were calculated from McNemar's Chi-square test to examine whether CHE incidence differed between 2 years in the total sample, or Chi-square test for independent samples to examine whether CHE incidence differed by frailty status in each year. **(A)** CHE incidence in total sample. **(B)** CHE incidence in particiants according to frailty status.

Figure 1 presents the incidence of CHE in the total sample and according to frailty status from 2011 to 2013. The CHE incidence among community-dwelling older adults in China was 21.76% in 2011 and increased to 26.46% in 2013. More specifically, the CHE incidence equaled 31.52, 23.66, and 19.58% in 2011 for frail, prefrail, and non-frail individuals, respectively, and it was 32.69, 28.75, and 24.01% in 2013 for frail, prefrail, and non-frail individuals, respectively. Prefrail and frail adults had a significantly higher CHE incidence than those who were robust in both 2011 and 2013 (*p* < 0.05).

### Longitudinal Relationship Between Frailty and CHE

Results for the longitudinal association between frailty and CHE among community-dwelling Chinese older adults are displayed in Table 2. When we examined frailty as a continuous variable, every one-component increase in frailty was found to significantly increase the risk for CHE in all models adjusting for covariates hierarchically (crude model: OR = 1.23, 95% CI = 1.13–1.34; fully-adjusted model: OR = 1.21, 95% CI = 1.11–1.32). Compared with non-frail individuals, prefrail or frail adults were associated with higher odds for CHE after controlling for age, gender, residence, education, marriage, income, health insurance, smoking, drinking, and comorbidity (prefrail: OR = 1.32, 95% CI = 1.14–1.52; frail: OR = 1.67, 95% CI = 1.13–2.47). Besides, we observed that three frailty components including weakness, exhaustion, and shrinking contributed to significantly increased likelihood of CHE (weakness: OR = 1.36, 95% CI = 1.14–1.63; exhaustion: OR = 1.60, 95% CI = 1.25–2.04; shrinking: OR = 1.29, 95% CI = 1.08–1.55) after controlling for the full list of predefined confounders, while the other two components including slowness and inactivity showed non-significant effect (slowness: OR = 1.08, 95% CI = 0.90–1.30; inactivity: OR = 1.03, 95% CI = 0.78–1.37). The above results remained all similar in models with adjustment for fewer covariates.

**Table 2:** Longitudinal association between frailty and catastrophic health expenditure (CHE) in community-dwelling Chinese older adults (*N* = 3,277).

**Variables**	**Catastrophic health expenditure, OR (95%CI)**				
	**Model 1^a^**	**Model 2^b^**	**Model 3^c^**	**Model 4^d^**	**Model 5^e^**
Every one-component increase in frailty	1.23 (1.13–1.34)***	1.23 (1.13–1.34)***	1.26 (1.15–1.37)***	1.25 (1.14–1.36)***	1.21 (1.11–1.32)***
Frailty phenotype					
Non-frail	1.00	1.00	1.00	1.00	1.00
Pre-frail	1.35 (1.17–1.55)***	1.34 (1.17–1.55)***	1.38 (1.20–1.59)***	1.36 (1.18–1.57)***	1.32 (1.14–1.52)***
Frail	1.75 (1.20–2.57)**	1.73 (1.18–2.55)**	1.83 (1.24–2.70)**	1.78 (1.21–2.64)**	1.67 (1.13–2.47)**
Frailty phenotype components					
Slowness (ref: no slowness)	1.10 (0.92–1.32)	1.09 (0.91–1.31)	1.13 (0.94–1.36)	1.12 (0.93–1.34)	1.08 (0.90–1.30)
Weakness (ref: no weakness)	1.40 (1.17–1.67)***	1.39 (1.16–1.67)***	1.44 (1.20–1.73)***	1.41 (1.18–1.69)***	1.36 (1.14–1.63)***
Exhaustion (ref: no exhaustion)	1.55 (1.21–1.98)***	1.55 (1.21–1.98)***	1.72 (1.34–2.20)***	1.70 (1.33–2.18)***	1.60 (1.25–2.04)***
Inactivity (ref: no inactivity)	1.06 (0.80–1.40)	1.05 (0.79–1.39)	1.02 (0.76–1.35)	1.02 (0.76–1.35)	1.03 (0.78–1.37)
Shrinking (ref: no shrinking)	1.35 (1.13–1.62)**	1.34 (1.12–1.61)**	1.31 (1.09–1.57)**	1.32 (1.10–1.59)**	1.29 (1.08–1.55)**

a *Model 1 was a crude model without adjustment for any covariates*.

b *Model 2 was adjusted for covariates including age and gender*.

c *Model 3 was adjusted for covariates including age, gender, residence, education, marital status, income, and health insurance*.

d *Model 4 was adjusted for covariates including age, gender, residence, education, marital status, income, health insurance, smoking, and drinking*.

e *Model 5 was adjusted for covariates including age, gender, residence, education, marital status, income, health insurance, smoking, drinking, and physical comorbidity*.

** *p < 0.01*,

*** *p < 0.001; OR, odds ratio; CI, confidence interval*.

### Stratification Analyses

**Figure 2:**
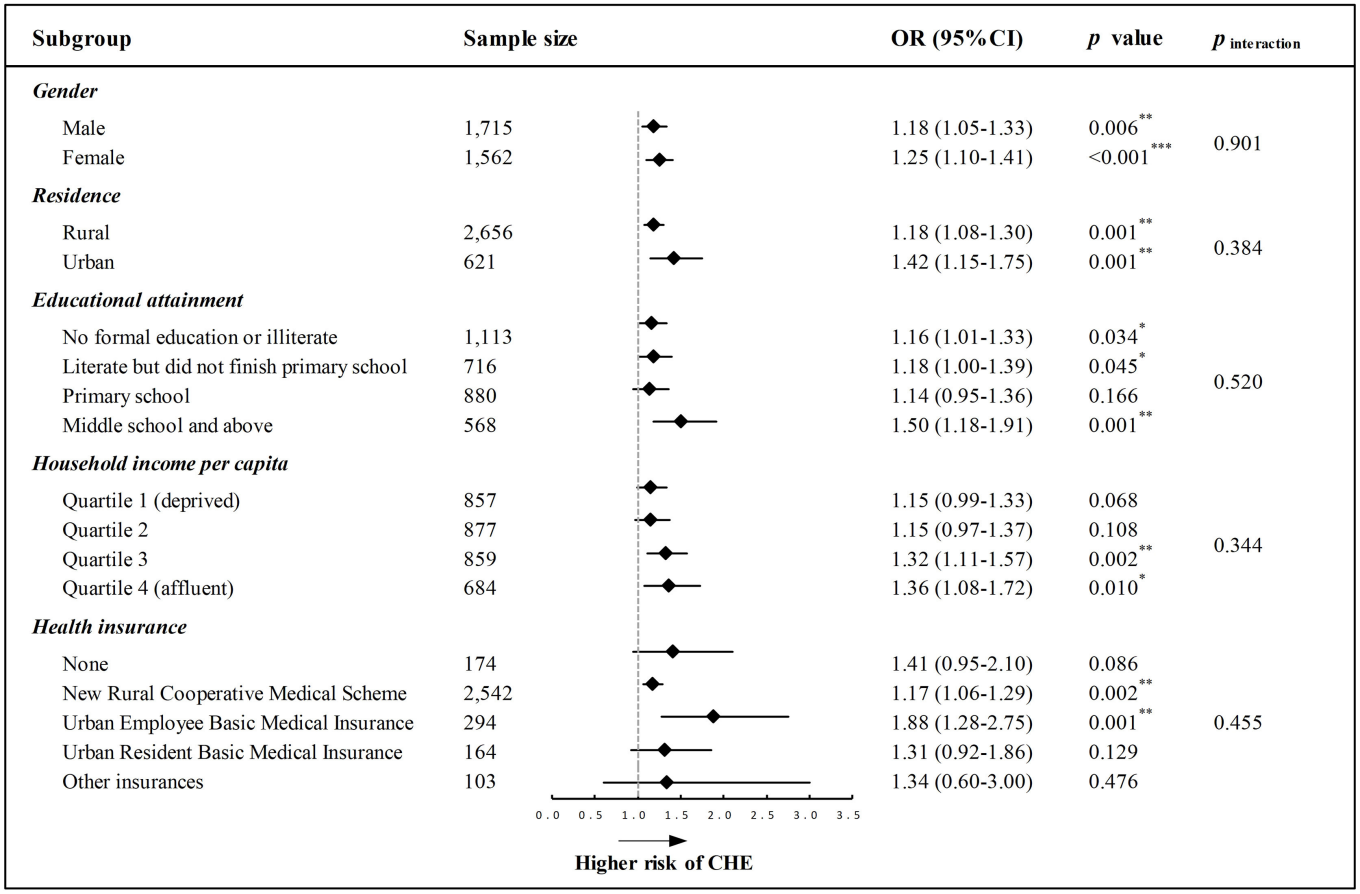
Forest plot depicting the longitudinal association between every one-component increase in frailty and CHE across different socioeconomic-related subgroups. All models were adjusted for the predefined full list of covariates (i.e., age, gender, residence, education, marital status, income, health insurance, smoking, drinking, and physical comorbidity) except the stratification variable. *p* for interaction (*p*_interaction_) was examined using the likelihood-ratio test. OR, odds ratio; CI, confidence interval; *p < 0.05, **p < 0.01, ***p < 0.001.

We further conducted stratification analyses to examine whether the effect of a one-component increase in frailty on CHE was varied by different socioeconomic-related subgroups, and the results are illustrated in Figure 2. The forest plot indicated that a one-component increase in frailty was associated with a higher likelihood for CHE after adjusting for the aforementioned covariates, and such a pattern of effect was similarly observed across different subgroups with varied gender, place of residence, educational attainment, household income level, and social health insurance (Figure 2). Results from the likelihood-ratio test supported that the interaction effect was all non-significant, indicating that the effect of frailty on CHE was comparable irrespective of gender, residence, education, household income, and health insurance (all *p* for interaction > 0.05).

### Sensitivity Analysis

Sensitivity analyses were carried out to test the robustness of our results, by using the different thresholds for classifying CHE (Table 3). The results were all consistent with the main findings when we defined CHE as ≥ 25% of the total non-food household expenditure, ≥ 10 and ≥ 25% of the total household expenditure.

**Table 3:** Longitudinal association between frailty and catastrophic health expenditure with different thresholds (*N* = 3,277).

**Variables**	**Catastrophic health expenditure with different thresholds, OR (95%CI)**					
	**Threshold 1:** **≥25% of households' total** **non–food expenditure**		**Threshold 2:** **≥25% of households' total expenditure**		**Threshold 3:** **≥10% of households' total expenditure**	
	**Model 1^a^**	**Model 2^b^**	**Model 1^a^**	**Model 2^b^**	**Model 1^a^**	**Model 2^b^**
Every one–component increase in frailty	1.25 (1.15–1.36)***	1.24 (1.14–1.35)***	1.29 (1.18–1.40)***	1.27 (1.16–1.38)***	1.27 (1.17–1.38)***	1.26 (1.16–1.37)***
Frailty phenotype						
Non–frail	1.00	1.00	1.00	1.00	1.00	1.00
Pre–frail	1.35 (1.18–1.55)***	1.33 (1.16–1.53)***	1.43 (1.24–1.66)***	1.40 (1.20–1.62)***	1.43 (1.24–1.64)***	1.40 (1.22–1.61)***
Frail	1.79 (1.22–2.63)**	1.75 (1.19–2.57)**	2.09 (1.42–3.08)***	2.01 (1.36–2.97)***	1.69 (1.14–2.48)**	1.66 (1.13–2.45)*
Frailty phenotype components						
Slowness (ref: no slowness)	1.08 (0.90–1.29)	1.06 (0.89–1.27)	1.14 (0.95–1.38)	1.13 (0.93–1.36)	1.16 (0.97–1.38)	1.14 (0.96–1.37)
Weakness (ref: no weakness)	1.39 (1.17–1.67)***	1.37 (1.14–1.63)***	1.50 (1.25–1.81)***	1.46 (1.21–1.75)***	1.40 (1.17–1.68)***	1.37 (1.15–1.64)***
Exhaustion (ref: no exhaustion)	1.66 (1.30–2.12)***	1.72 (1.34–2.19)***	1.65 (1.29–2.11)***	1.69 (1.31–2.16)***	1.61 (1.26–2.05)***	1.67 (1.30–2.13)***
Inactivity (ref: no inactivity)	1.12 (0.84–1.48)	1.11 (0.83–1.48)	1.08 (0.80–1.46)	1.06 (0.78–1.44)	1.09 (0.82–1.45)	1.08 (0.81–1.44)
Shrinking (ref: no shrinking)	1.37 (1.14–1.64)***	1.33 (1.11–1.59)**	1.39 (1.15–1.68)***	1.34 (1.11–1.62)**	1.41 (1.18–1.69)***	1.38 (1.15–1.65)***

a *Model 1 was adjusted for covariates including age and gender*.

b *Model 2 was adjusted for covariates including age, gender, residence, education, marital status, income, health insurance, smoking, drinking, and physical comorbidity*.

* *p < 0.05*,

** *p < 0.01*,

*** *p < 0.001; OR, odds ratio; CI, confidence interval*.

## Discussions

To our knowledge, this is the first attempt to investigate the longitudinal association of frailty with CHE among older people. The major strength of our study includes the cohort study design that enables us to examine the temporal relationship, its relatively large sample size from a nationwide community-based population, and the exploration into a topic that is not adequately addressed. In our sample, the incidence of CHE at the 40% threshold among community-dwelling older adults was 21.76% in 2011 and increased to 26.46% in 2013. The increasing trend of CHE prevalence was similarly observed in other Chinese studies, which may be partly explained by the rising OOP healthcare costs over time along with the absence of effective measures to cut down expenditures or share financial risks for individuals (3, 23). Several important findings are drawn from this study as follows.

The main finding of our study is that frailty appears to predict increased risk for CHE among community-dwelling older adults in China, suggesting the substantial burden from frailty on affecting the overall quality of household living standards. The majority of previous literature showed consistent findings suggesting that health disorders, such as chronic diseases (2, 24, 25), cancer (26), disability (27, 28), injuries (29), and depression (30), were associated with healthcare-related financial catastrophe, even though they rarely studied the impact from frailty. Only two studies were identified in terms of the association between CHE and frailty, which yet yielded inconclusive results (18, 19). That is, one study conducted by Jing et al. (18) demonstrated that the co-occurrence of frailty increased the risk of incurring CHE based on a sample of 606 single empty-nest elderly with multimorbidity (18), while another study from Gao et al. (19) suggested that frailty was not significantly associated with CHE among 5,204 community-dwelling adults aged at least 60 years (19). Both studies were, however, limited in their cross-sectional study design that failed to determine the chronological sequence of events, so the present study was advantageous by adopting a panel data analysis that could lead to more convincing results. The discrepancies in the observed association between frailty and CHE may be owing to the variations in the studied population, study design, and sample size across studies. Our finding that frailty could be a catastrophic condition is unsurprising given the following speculations: first, frailty is often associated with health decline and productivity loss, resulting in an inability to earn income (11, 12, 18); second, frail individuals are often found to incur higher medical expenditure due to more intensive health service use and heavier dependency on continuing care following the hospital discharge, which could eventually increase the likelihood of suffering from CHE (14–17).

Among the five FP components, the onset of weakness, exhaustion, and shrinking were found to be associated with CHE in this study, whereas slowness and inactivity were not significantly related to CHE. Our finding adds to the existing literature by comprehensively ascertaining the influence of each frailty component on CHE for the first time. Despite the lack of available research investigating the impacts of FP components on CHE, accumulating evidence has attempted to figure out their relationships with healthcare costs (31–33). For example, a cross-sectional study among 2,598 older participants from Germany showed that only weight loss and exhaustion were significantly associated with total healthcare costs (31). Another longitudinal study in Germany indicated that the onset of exhaustion was the only symptom associated with an increase in total healthcare costs (32). Ensrud et al. (33, 34) conducted cohort studies among older women and men separately in the United States and observed that each frailty component was associated with higher total costs (33, 34). The findings are still scarce and inconsistent so that further attention is necessary to improve the identification of high-risk older adults through frailty symptom assessment.

This study additionally demonstrated that the relationship between an increasing level of frailty and CHE remained consistent irrespective of socioeconomic-related differences with regard to gender, residence, education, household income, and health insurance. This contrasted with one previous research (18) suggesting that frail people with poor economic status were more likely to incur CHE than those with higher economic status, but their study population was limited to single empty-nest elderly with multimorbidity in one province of rural China. However, another research investigating physical multimorbidity and CHE revealed similar findings as in this study, indicating that the effect of comorbidity on CHE persisted among different household economic levels and across all health insurance programs (2). Other prior studies have only examined the disparities in healthcare utilization or costs associated with frailty rather than CHE, and their results were also controversial (14, 35, 36). For instance, some studies found gender interaction in inpatient use (36) or outpatient payment (35), whereas other studies identified no gender interaction in healthcare use (14) or total healthcare payment (35). Overall, the moderation effect of socioeconomic-related factors is not yet well-understood to date, warranting further large-scale and longitudinal investigations.

Findings from the present study have important practical implications. Frailty is increasingly prevalent and has emerged as an independent risk factor for healthcare-related financial catastrophe among community-dwelling older adults, indicating that early screening or assessment of frailty in the community setting may assist with identifying the targeted population at high risk of being reduced to poverty by healthcare costs. Policy-makers, clinicians, or public health authorities shall raise awareness about the increasing burden that frailty will pose on the healthcare system as well as the substantial benefit that proactive efforts to address frailty will bring to alleviate economic burden or inequalities among older individuals. Older people themselves should also be empowered with adequate knowledge and skills to prevent or reverse frailty, such as the capacity in detecting early warning signs of frailty and responsibility in modifying their unhealthy lifestyles.

Several limitations should also be taken into consideration. First, our measurement of CHE considered only the incurred outpatient and inpatient health costs. There could be healthcare spending from other sources that are not examined, so that the actual rate of CHE may be underestimated. Second, this study collected the key information *via* self-report instead of using clinical or objective measures, thus the results could be affected by recall bias. Third, due to the unavailability of whole-year data regarding outpatient expenditure, this study extrapolated monthly costs to the entire year instead to obtain the estimation of annual spending. This approach was yet not precise, and interpretation of the results would thus require caution.

## Conclusion

In conclusion, frailty is found to be a significant predictor for CHE among the community-dwelling older adults in China, and such effect remains similar irrespective of socioeconomic-related factors including gender, residence, education, household income, and health insurance. This study sheds light on the financial catastrophe associated with the increasingly recognized public health priority of frailty. We provide scientific evidence for policy-makers to develop cost-effective strategies for community-based early prevention and management of frailty among the older population, as well as to improve the health insurance scheme and healthcare financing system to further facilitate more accessible and affordable health services.

## Data Availability Statement

The publicly available datasets were analyzed in this study, which can be found in the link: http://charls.pku.edu.cn/index/en.html.

## Ethics Statement

The studies involving human participants were reviewed and approved by the Ethics Review Committee of Peking University (No.: IRB00001052-11015). The patients/participants provided their written informed consent to participate in this study.

## Author Contributions

LF and WD contributed to the conception and design of the study. LF, YL, and SC conducted the data analyses. LF drafted the manuscript. All authors reviewed and approved the manuscript before submission.

## Funding

This work was supported by the Natural Science Foundation of China (NSFC 71704192), the Department of Education of China (No. 1125000172), the Fundamental Research Funds for the Central Universities (2242021R41104, 2242021S40011, 2242020R10007, 3225002002A1), and the Zhishan Youth Scholar Program of Southeast University (2019–2021). The funders had no role in study design, data collection and analysis, preparation and review of the manuscript, or decision to submit the manuscript for publication.

## Conflict of Interest

The authors declare that the research was conducted in the absence of any commercial or financial relationships that could be construed as a potential conflict of interest.

## Publisher's Note

All claims expressed in this article are solely those of the authors and do not necessarily represent those of their affiliated organizations, or those of the publisher, the editors and the reviewers. Any product that may be evaluated in this article, or claim that may be made by its manufacturer, is not guaranteed or endorsed by the publisher.

## References

[1] Evans DB Etienne C. Health systems financing and the path to universal coverage. Bull World Health Organ. (2010) 88:402. 10.2471/BLT.10.078741 20539847 PMC2878164

[2] Zhao Y Atun R Oldenburg B McPake B Tang SL Mercer SW. Physical multimorbidity, health service use, and catastrophic health expenditure by socioeconomic groups in China: an analysis of population-based panel data. Lancet Glob Health. (2020) 8:e840–9. 10.1016/S2214-109X(20)30127-3 32446349 PMC7241981

[3] Liu S Coyte PC Fu M Zhang Q. Measurement and determinants of catastrophic health expenditure among elderly households in China using longitudinal data from the CHARLS. Int J Equity Health. (2021) 20:62. 10.1186/s12939-020-01336-8 33608014 PMC7893946

[4] Cylus J Thomson S Evetovits T. Catastrophic health spending in Europe: equity and policy implications of different calculation methods. Bull World Health Organ. (2018) 96:599–609. 10.2471/BLT.18.209031 30262941 PMC6154073

[5] Wagstaff A Flores G Hsu J Smitz MF Chepynoga K Buisman LR. Progress on catastrophic health spending in 133 countries: a retrospective observational study. Lancet Glob Health. (2018) 6:e169–79. 10.1016/S2214-109X(17)30429-1 29248367

[6] Kasahun GG Gebretekle GB Hailemichael Y Woldemariam AA Fenta TG. Catastrophic healthcare expenditure and coping strategies among patients attending cancer treatment services in Addis Ababa, Ethiopia. BMC Public Health. (2020) 20:984. 10.1186/s12889-020-09137-y 32571275 PMC7310089

[7] Dent E Lien C Lim WS Wong WC Wong CH Ng TP. The Asia-Pacific clinical practice guidelines for the management of frailty. J Am Med Dir Assoc. (2017) 18:564–75. 10.1016/j.jamda.2017.04.018 28648901

[8] Dent E Martin FC Bergman H Woo J Romero-Ortuno R Walston JD. Management of frailty: opportunities, challenges, and future directions. Lancet. (2019) 394:1376–86. 10.1016/S0140-6736(19)31785-4 31609229

[9] Wu CK Smit E Xue QL Odden MC. Prevalence and correlates of frailty among community-dwelling Chinese older adults: the China Health and Retirement Longitudinal Study. J Gerontol A Biol Sci Med Sci. (2017) 73:102–8. 10.1093/gerona/glx098 28525586 PMC5861883

[10] HeBMaYWangCJiangMGengCChangX. Prevalence and risk factors for frailty among community-dwelling older people in China: a systematic review and meta-analysis. J Nutr Health Aging. (2019) 23:442–50. 10.1007/s12603-019-1179-931021361

[11] Vermeiren S Vella-Azzopardi R Beckwee D Habbig AK Scafoglieri A Jansen B. Frailty and the prediction of negative health outcomes: a meta-analysis. J Am Med Dir Assoc. (2016) 17:1163. e1-1163. e17. 10.1016/j.jamda.2016.09.010 27886869

[12] Liu ZY Wei YZ Wei LQ Jiang XY Wang XF Shi Y. Frailty transitions and types of death in Chinese older adults: a population-based cohort study. Clin Interv Aging. (2018) 13:947–56. 10.2147/CIA.S157089 29805253 PMC5960243

[13] Xu WH Li YX Hu YX Wu CK. Association of frailty with recovery from disability among community-dwelling Chinese older adults: China health and retirement longitudinal study. BMC Geriatr. (2020) 20:119. 10.1186/s12877-020-01519-6 32228463 PMC7106588

[14] Fan LJ Tian Y Wang JW Ding Y Wang SY Xue H. Frailty predicts increased health care utilization among community-dwelling older adults: a longitudinal study in China. J Am Med Dir Assoc. (2021) S1525–8610(21)00194-8. 10.1016/j.jamda.2021.01.082 33662331

[15] Kojima G. Increased healthcare costs associated with frailty among community-dwelling older people: a systematic review and meta-analysis. Arch Gerontol Geriatr. (2019) 84:103898. 10.1016/j.archger.2019.06.003 31228673

[16] Jin HY Liu XT Xue QL Chen S Wu CK. The association between frailty and healthcare expenditure among Chinese older adults. J Am Med Dir Assoc. (2020) 21:780–5. 10.1016/j.jamda.2020.03.008 32331768

[17] Chi J Chen F Zhang J Niu XD Tao HX Ruan HH. Impacts of frailty on health care costs among community-dwelling older adults: a meta-analysis of cohort studies. Arch Gerontol Geriatr. (2021) 94:104344. 10.1016/j.archger.2021.104344 33516075

[18] Jing ZY Li J Fu PP Wang Y Yuan YM Zhao D. Catastrophic health expenditure among single empty-nest elderly with multimorbidity in rural Shandong, China: the effect of co-occurrence of frailty. Int J Equity Health. (2021) 20:23. 10.1186/s12939-020-01362-6 33413429 PMC7792165

[19] Gao K Li BL Yang L Zhou D Ding KX Yan J. Cardiometabolic diseases, frailty, and healthcare utilization and expenditure in community-dwelling Chinese older adults. Sci Rep. (2021) 11:7776. 10.1038/s41598-021-87444-z 33833338 PMC8032763

[20] Zhao YH Hu YS Smith JP Strauss J Yang GH. Cohort profile: the China Health and Retirement Longitudinal Study (CHARLS). Int J Epidemiol. (2014) 43:61–8. 10.1093/ije/dys203 23243115 PMC3937970

[21] Fried LP Tangen CM Walston J Newman AB Hirsch C Gottdiener J. Frailty in older adults: evidence for a phenotype. J Gerontol A Biol Sci Med Sci. (2001) 56:M146–56. 10.1093/gerona/56.3.M146 11253156

[22] Si Y Zhou ZL Su M Ma M Xu YJ Heitner J. Catastrophic healthcare expenditure and its inequality for households with hypertension: evidence from the rural areas of Shaanxi Province in China. Int J Equity Health. (2017) 16:27. 10.1186/s12939-016-0506-6 28666448 PMC5493855

[23] Si Y Zhou ZL Su M Wang X Lan X Wang D. Decomposing inequality in catastrophic health expenditure for self-reported hypertension household in Urban Shaanxi, China from 2008 to 2013: two waves' cross-sectional study. BMJ Open. (2019) 9:e023033. 10.1136/bmjopen-2018-023033 31076467 PMC6528006

[24] Arsenijevic J Pavlova M Rechel B Groot W. Catastrophic health care expenditure among older people with chronic diseases in 15 European countries. PLoS ONE. (2016) 11:e0157765. 10.1371/journal.pone.0157765 27379926 PMC4933384

[25] Choi JW Choi JW Kim JH Yoo KB Park EC. Association between chronic disease and catastrophic health expenditure in Korea. BMC Health Serv Res. (2015) 15:26. 10.1186/s12913-014-0675-1 25608983 PMC4307618

[26] Zheng A Duan WJ Zhang L Bao XT Mao XY Luo Z. How great is current curative expenditure and catastrophic health expenditure among patients with cancer in China? A research based on “System of Health Account 2011.” *Cancer Med*. (2018) 7:4036–4043. 10.1002/cam4.1590 29923330 PMC6089193

[27] Lee JE Shin K Do YK Yang EJ. Catastrophic health expenditures for households with disabled members: evidence from the Korean Health Panel. J Korean Med Sci. (2016) 31:336–44. 10.3346/jkms.2016.31.3.336 26955233 PMC4779856

[28] Palmer M Nguyen T Neeman T Berry H Hull T Harley D. Health care utilization, cost burden and coping strategies by disability status: an analysis of the Viet Nam National Health Survey. Int J Health Plann Manage. (2011) 26:e151–68. 10.1002/hpm.1052 20583316

[29] Yadav J Menon G Agarwal A John D. Burden of injuries and its associated hospitalization expenditure in India. Int J Inj Contr Saf Promot. (2021) 28:153–61. 10.1080/17457300.2021.1879163 33557698

[30] Patel V Chisholm D Kirkwood BR Mabey D. Prioritizing health problems in women in developing countries: comparing the financial burden of reproductive tract infections, anaemia and depressive disorders in a community survey in India. Trop Med Int Health. (2007) 12:130–9. 10.1111/j.1365-3156.2006.01756.x 17207157

[31] Bock JO Konig HH Brenner H Haefeli WE Quinzler R Matschinger H. Associations of frailty with health care costs - results of the ESTHER cohort study. BMC Health Serv Res. (2016) 16:128. 10.1186/s12913-016-1360-3 27074800 PMC4831082

[32] Hajek A Bock JQ Saum KU Matschinger H Brenner H Holleczek B. Frailty and healthcare costs-longitudinal results of a prospective cohort study. Age Ageing. (2018) 47:233–41. 10.1093/ageing/afx157 29036424

[33] Ensrud KE Kats AM Schousboe JT Taylor BC Cawthon PM Hillier TA. Frailty phenotype and healthcare costs and utilization in older women. J Am Geriatr Soc. (2018) 66:1276–83. 10.1111/jgs.15381 29684237 PMC6097947

[34] Ensrud KE Kats AM Schousboe JT Taylor BC Vo TN Cawthon PM. Frailty phenotype and healthcare costs and utilization in older men. J Am Geriatr Soc. (2020) 68:2034–42. 10.1111/jgs.16522 32402097 PMC7666024

[35] Li CY Snih SA Chou LN Karmarkar A Kuo YF Markides KS. Frailty transitions predict healthcare use and Medicare payments in older Mexican Americans: a longitudinal cohort study. BMC Geriatr. (2020) 20:189. 10.1186/s12877-020-01583-y 32487037 PMC7268381

[36] Zhang J Xu LZ Sun L Li JJ Qin WZ. Gender difference in the association of frailty and health care utilization among Chinese older adults: results from a population-based study. Aging Clin Exp Res. (2020) 32:1985–91. 10.1007/s40520-019-01410-4 31745830

